# Determining the role of genetic risk scores in symptomatic cancer detection

**DOI:** 10.3399/bjgp23X732069

**Published:** 2023-02-24

**Authors:** Sarah ER Bailey, Celia AM Butler, Evangelos Katsampouris, Larry Kessler, Samantha L Quaife, Sibel Saya, Samuel WD Merriel

**Affiliations:** DISCOVERY Group, University of Exeter Medical School, University of Exeter, Exeter, UK.; DISCOVERY Group, University of Exeter Medical School, University of Exeter, Exeter, UK.; Wolfson Institute of Population Health, Barts and The London School of Medicine and Dentistry, Queen Mary University of London, London, UK.; Department of Health Systems and Population Health, School of Public Health, University of Washington, Seattle, US.; Wolfson Institute of Population Health, Barts and The London School of Medicine and Dentistry, Queen Mary University of London, London, UK.; University of Melbourne Centre for Cancer Research, Victorian Comprehensive Cancer Centre; Department of General Practice, University of Melbourne, Victoria, Australia.; GP and National Institute for Health and Care Research Academic Clinical Lecturer, Centre for Primary Care and Health Services Research, University of Manchester, Manchester.

## INTRODUCTION

Improving cancer diagnosis is a national priority in the UK, with the *NHS Long Term Plan* pledging to increase the percentage of cancers found at an early stage from 50% to 75% by 2028.^1^ Patients with cancer diagnosed at an early stage generally have better outcomes and longer survival. Most cancers in the UK are diagnosed following a symptomatic presentation to primary care, with over 80% of patients with cancer seeing their GP in the year before diagnosis.^2^ National screening programmes are available for breast, colorectal, and cervical cancer, but identify only 5% of cases.^3^ A lung cancer screening programme has recently been approved in the UK.

GPs select patients for referral based on presenting clinical features; individual or combinations of features representing a 3% or greater chance of cancer should trigger urgent investigation.^4^ For patients with features in the 1%–2% risk category, triage tests to further inform clinical judgement include general or cancer-specific blood tests, imaging, or faecal immunochemical tests, which identify haemoglobin in a faeces sample. National Institute for Health and Care Excellence (NICE) guidance NG12 recommends certain investigations and referrals based on the suspected site of malignancy;^4^ those recommendations are based on clinical features alone, and do not account for genetic risk of cancer or any other factors that make cancer more likely to develop (other than age, which is used to stratify some of the recommendations). Although GPs can take family history in a consultation, the best available proxy for genetic risk of cancer, NG12 recommends the same course of action irrespective of an individual patient’s family history of malignancy.

## GENETIC RISK SCORES

Since the human genome was first sequenced in 2008, genome-wide association studies (GWAS) have identified individual genetic variants, known as single nucleotide polymorphisms (SNPs), which are associated with the risk of cancer.^5^ Individually, each SNP contributes a very small increase in risk, but the predictive power of multiple SNPs can be combined into one clinically useful genetic risk score (GRS).^6^ A GRS is the sum of risk alleles an individual carries, weighted by each SNP’s predictive strength. It gives a personalised risk of developing the disease of interest in an individual’s lifetime. GRSs for cancer have been shown to increase disease predictions over and above family history of cancer alone, and perform strongest in cancers with a high degree of heritability.^7^ There is now a substantial and consistently growing body of evidence for their clinical use in the early detection of cancer; however, this is almost exclusively in the context of asymptomatic risk prediction and targeted screening, and is limited to heritable cancers. A recent study showed that a prostate cancer GRS could improve risk stratification in primary care.^8^

Most strategies to improve early cancer detection in primary care involve improving the selection of patients for referral and investigation, introducing new triage tests, or encouraging earlier symptom recognition in patients and clinicians. Incorporating genetic risk data into primary care- suspected cancer risk assessments, in the form of GRS, is an as-yet underresearched area that could improve risk stratification and inform clinical decision making for those patients who do present. Given primary care services in high-income countries such as the UK already use electronic health records (EHRs), the integration of this additional clinical information into practice is feasible. Patient data held in EHRs can be accessed during a GP consultation; this includes birth date, sex, ethnicity, body mass index, as well as diagnosed conditions, test results, and prescriptions. Once sequenced, an individual’s genomic data (whether the full genome or only those parts required to derive the GRS) could be held on file alongside other patient factors, for easy access during a consultation, and incorporation into disease risk scores, such as cancer risk assessment tools^9^^,^^10^ and QCancer.^11^ It is possible that the incorporation of a GRS, along with other baseline risk variables, into clinical testing pathways for those with possible cancer symptoms in primary care will result in more effective triaging and therefore better early detection of cancer.

## LOOKING TO THE FUTURE

Although full genome sequencing is not currently available in primary care, we are fast moving towards a future in which this is more widely available; the *NHS Long Term Plan* pledges that the NHS will be the first national health service to offer full genome sequencing as standard across the population, possibly even at birth (although this suggestion comes with its own unique set of moral and ethical considerations that would be imperative to understand). The Cancer Research UK Stratified Medicine Programme 2, a UK-wide genomic screening programme, has demonstrated that routine genomic testing can be delivered at scale in a timely manner within the NHS.^12^ As these changes approach, research is urgently needed to fully explore not only the full range of potential benefits but also the potential implications of the wider implementation of genomic sequencing and the use of genetic data in health care. This must be underpinned by a stakeholder- driven agenda to ensure the prioritisation of questions integral to equitable, ethical, feasible (from professional/health service), and patient-centred implementation. A particular issue is the lack of a complete understanding of the natural history of many cancers and whether a genetically driven algorithm that assists in earlier identification of cancers is producing a positive net health benefit for the population.

The patient and public voice must steer the potential implementation of GRSs and genetic information into primary care. The potential psychosocial and behavioural impacts of genome testing in the context of cancer screening have been explored;^13^ however, it is not known how this would translate for those who present to primary care with possible symptoms of an underlying cancer. Safe storage of genetic data is paramount to maintain public trust in the health service and research utilising this information to improve primary care diagnosis and treatment.

To address these issues, researcher access to large, linked datasets that are representative of the UK population is crucial. Currently, the UK Biobank (UKBB) is the only available source of genomic data linked to the primary care record and is not representative of the general population.^14^ This has the potential to have a negative impact on the credibility and suitability of the resulting interventions developed and studied within homogeneous populations among groups underrepresented in development. Those who are most disadvantaged in cancer diagnosis are generally underrepresented in genetic research and UKBB is no exception. This must change to ensure that progress in genomic testing is equitable and does not further increase inequalities in cancer mortality.

In summary, there is potential for improving cancer detection strategies in those who present with symptoms with genetic risk scores. Widespread genomic testing is coming. The research community needs to act now not only to model and understand the full gamut of potential benefits but also the implications of this for cancer diagnosis using inclusive and representative methodologies, and in close collaboration with lay and clinical stakeholders.
